# Complete genome sequence of *Beutenbergia cavernae* type strain (HKI 0122^T^)

**DOI:** 10.4056/sigs.1162

**Published:** 2009-07-20

**Authors:** Miriam Land, Rüdiger Pukall, Birte Abt, Markus Göker, Manfred Rohde, Tijana Glavina Del Rio, Hope Tice, Alex Copeland, Jan-Fang Cheng, Susan Lucas, Feng Chen, Matt Nolan, David Bruce, Lynne Goodwin, Sam Pitluck, Natalia Ivanova, Konstantinos Mavromatis, Galina Ovchinnikova, Amrita Pati, Amy Chen, Krishna Palaniappan, Loren Hauser, Yun-Juan Chang, Cynthia C. Jefferies, Elizabeth Saunders, Thomas Brettin, John C. Detter, Cliff Han, Patrick Chain, James Bristow, Jonathan A. Eisen, Victor Markowitz, Philip Hugenholtz, Nikos C. Kyrpides, Hans-Peter Klenk, Alla Lapidus

**Affiliations:** 1 DOE Joint Genome Institute, Walnut Creek, California, USA; 2 Oak Ridge National Laboratory, Oak Ridge, Tennessee, USA; 3 DSMZ - German Collection of Microorganisms and Cell Cultures GmbH, Braunschweig, Germany; 4 HZI - Helmholtz Centre for Infection Research, Braunschweig, Germany; 5 Los Alamos National Laboratory, Bioscience Division, Los Alamos, New Mexico USA; 6 Biological Data Management and Technology Center, Lawrence Berkeley National Laboratory, Berkeley, California, USA; 7 Lawrence Livermore National Laboratory, Livermore, California, USA; 8 University of California Davis Genome Center, Davis, California, USA

**Keywords:** mesophile, non-pathogenic, aerobic and microaerophilic, rod-coccus growth cycle, MK-8(H_4_), actinomycete, *Micrococcineae*

## Abstract

*Beutenbergia cavernae* (Groth *et al.* 1999) is the type species of the genus and is of phylogenetic interest because of its isolated location in the actinobacterial suborder *Micrococcineae. B. cavernae* HKI 0122^T^ is a Gram-positive, non-motile, non-spore-forming bacterium isolated from a cave in Guangxi (China). *B. cavernae* grows best under aerobic conditions and shows a rod-coccus growth cycle. Its cell wall peptidoglycan contains the diagnostic L-lysine ← L-glutamate interpeptide bridge. Here we describe the features of this organism, together with the complete genome sequence, and annotation. This is the first completed genome sequence from the poorly populated micrococcineal family *Beutenbergiaceae*, and this 4,669,183 bp long single replicon genome with its 4225 protein-coding and 53 RNA genes is part of the *** G****enomic* *** E****ncyclopedia of* *** B****acteria and* *** A****rchaea * project.

## Introduction

Beutenbergia cavernae strain HKI 0122^T^ (DSM 12333 = ATCC BAA-8 = JCM 11478) is the type strain of the species, which represents the type species of the genus Beutenbergia, the type genus of the family Beutenbergiaceae [1]. B. cavernae was described by Groth et al. 1999 as Gram-positive, non-motile and non-spore-forming [1].

**Figure 1:**
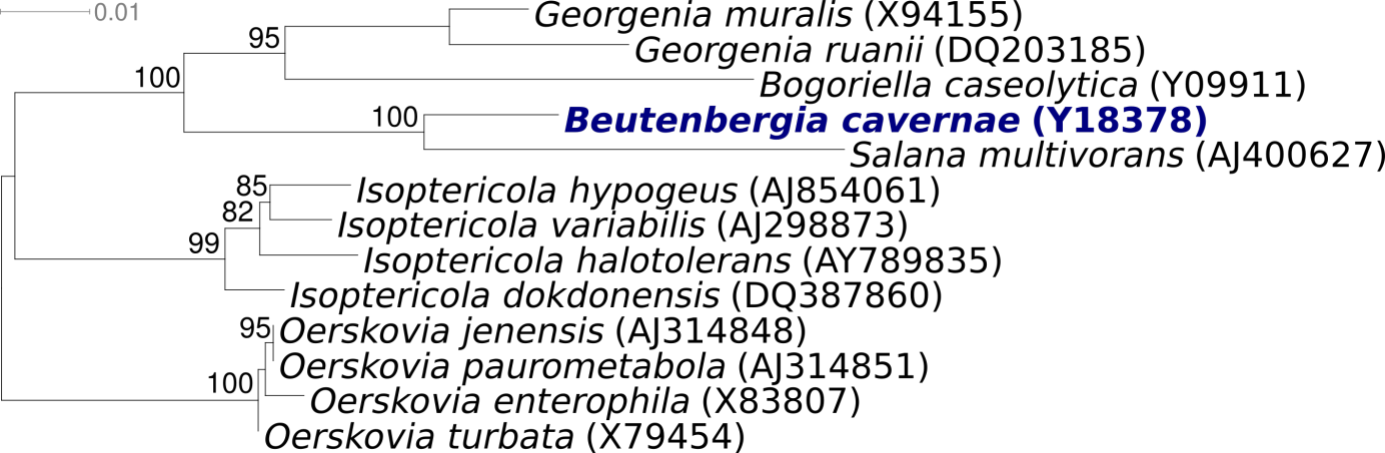
Phylogenetic tree of *B. cavernae* HKI 0122^T^ and all type strains of the genus *Beutenbergia*, inferred from 1411 aligned characters [5,6] of the 16S rRNA sequence under the maximum likelihood criterion [7]. The tree was rooted with species from the genera *Isoptericola* and *Oerskovia*, both also members of the actinobacterial suborder *Micrococcineae*. The branches are scaled in terms of the expected number of substitutions per site. Numbers above branches are support values from 1000 bootstrap replicates if larger than 60%. Strains with a genome-sequencing project registered in GOLD [8] are printed in blue; published genomes in bold.

The organism is of significant interest for its position in the tree of life within the small (2 type strains) family Beutenbergiaceae Zhi, et al, 2009 emend. Schumann et al. 2009 in the actinobacterial suborder Micrococcineae [2], which in addition to the genus Beutenbergia contains only the genus Salana [3,4] (Figure 1), also otherwise stated in a recent overview on the class Actinobacteria [2]. Here we present a summary classification and a set of features for B. cavernae strain HKI 0122^T^ (Table 1), together with the description of the complete genome sequencing and annotation.

**Table 1:** Classification and general features of *B. cavernae* HKI 0122^T^ based on the MIGS recommendations [9]

**MIGS ID**	**Property**	**Term**	**Evidence code**
	Current classification	Domain *Bacteria*	
		Phylum *Actinobacteria*	
		Class *Actinobacteria*	TAS [10]
		Order *Actinomycetales*	TAS [10]
		Suborder *Micrococcineae*	TAS [2]
		Family *Beutenbergiaceae*	TAS [2]
		Genus *Beutenbergia*	TAS [1]
		Species *Beutenbergia cavernae*	TAS [1]
		Type strain HKI 0122	
	Gram stain	positive	TAS [1]
	Cell shape	varies; rod-coccus growth cycle	TAS [1]
	Motility	nonmotile	TAS [1]
	Sporulation	non-sporulating	TAS [1]
	Temperature range	mesophile	TAS [1]
	Optimum temperature	28°C	TAS [1]
	Salinity	tolerance of 2-4% (w/v) NaCl	TAS [1]
MIGS-22	Oxygen requirement	aerobic and microaerobic, no growth under anaerobic conditions	TAS [1]
	Carbon source	glucose, maltose, mannose, cellobiose	TAS [1]
	Energy source	unknown	
MIGS-6	Habitat	cave (soil)	TAS [1]
MIGS-15	Biotic relationship		
MIGS-14	Pathogenicity	none	NAS
	Biosafety level	1	TAS [11]
	Isolation	cave, soil between rocks	TAS [1]
MIGS-4	Geographic location	Guangxi, China	TAS [1]
MIGS-5	Sample collection time	about 1999	TAS [1]
MIGS-4.1 MIGS-4.2	Longitude Latitude	110.263306 25.307878	TAS [1]
MIGS-4.3	Depth	not reported	
MIGS-4.4	Altitude	not reported	

Evidence codes - IDA: Inferred from Direct Assay (first time in publication); TAS: Traceable Author Statement (i.e., a direct report exists in the literature); NAS: Non-traceable Author Statement (i.e., not directly observed for the living, isolated sample, but based on a generally accepted property for the species, or anecdotal evidence). These evidence codes are from the Gene Ontology project [12]. If the evidence code is IDA the property was directly observed for a live isolate by one of the authors or an expert mentioned in the acknowledgements.

In addition to strain HKI 0122^T^, only one additional strain (HKI 0132) was isolated from the soil sample collected in the Reed Flute Cave near Guilin, Guangxi, China. HKI 0132 was also classified in the species B. cavernae [1]. No closely related isolates and uncultivated clones with more than 97% 16S rRNA gene sequence identity are recorded in the microbiological literature, nor can any phylotype from environmental samples or genomic surveys be directly linked to B. cavernae.

**Figure 2:**
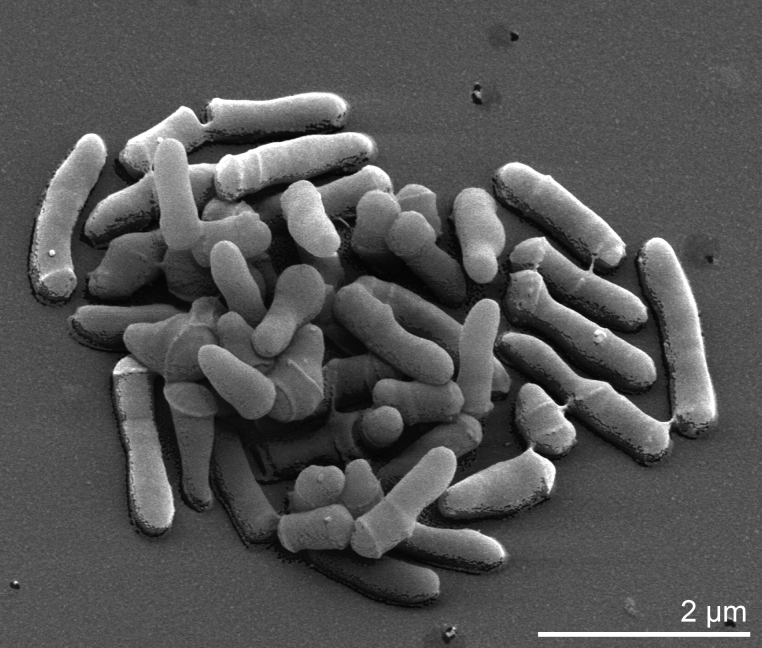
Scanning electron micrograph of *B. cavernae* HKI 0122^T^

B. cavernae cells vary in shape and colonies grown on rich medium vary in color from cream to bright yellow. In young cultures, cells are irregular rods arranged in palisades, clusters or in pairs at an angle to give V-formations (Figure 2) [1]. Cells in stationary cultures are predominantly coccoid, occurring singly, in pairs, irregular clusters and short chains. During growth in complex media a rod-coccus growth cycle was observed [1]. B. cavernae grow well under aerobic and microaerophilic conditions, but not under anaerobic conditions [1]. The optimal growth temperature is 28°C [1].

B. cavernae is able to degrade casein, esculin, gelatin and potato starch. Acids are produced from L-arabinose, D-cellobiose, dextrin, D-fructose, D-galactose, D-glucose, glycerol, inulin, maltose, D-mannose, D-raffinose, L-rhamnose, D-ribose, salicin, sucrose, starch, trehalose and D-xylose. There is no acid production from D-glucitol, lactose and D-mannitol. Nitrate is reduced to nitrite, H_2_S is produced [1].

## Classification and features

Figure 1. shows the phylogenetic neighborhood of B. cavernae strain HKI 0122^T^ in a 16S rRNA based tree. Analysis of the two identical 16S rRNA gene sequences in the genome of strain HKI differed by four nucleotides from the previously published 16S rRNA sequence generated from DSM 12333 (Y18378). The slight differences between the genome data and the reported 16S rRNA gene sequence is most likely due to sequencing errors in the previously reported sequence data .

### Chemotaxonomy

The peptidoglycan of B. cavernae HKI 0122^T^ contains D- and L-alanine, D- and L-glutamic acid and L-lysine, with the latter widely distributed among actinobacteria [1]. The strain possesses a type A4〈 peptidoglycan with a diagnostic LLys←L-Glu interpeptide bridge, type A11.54 according to http://www.dsmz.de/microorganisms/. Glucose, mannose and galactose are the cell wall sugars [1]. The fatty acid profile of strain B. cavernae HKI 0122^T^ is dominated by 13-methyl tetradecanoic (iso-C_15:0_; 43.7%) and 12-methyl tetradecanoic (anteiso-C_15:0_; 34.6%) saturated, branched chain acids. Other predominantly saturated fatty acids play a minor role in the cellular fatty acid composition of the strain: iso-C_14:0_ (0.9%), C_14:0_ (1.9%); C_15:0_ (0.9%) isoC_16:0_ (2.3%), C_16:0_ (6.8%), isoC_17:0_ (3.1%), anteiso-C_17:0_ (4.9%), und C_18:1_ (0.9%) [1]. Mycolic acids are not present [1]. MK-8(H_4_) is the major menaquinone, complemented by minor amounts of MK-8(H_2_), MK-8 and MK-9(H_4_) [1]. The combination of the LLys←L-Glu interpeptide bridge and MK-8(H_4_) as the dominating menaquinone is shared with the organisms from the neighboring genera Bogoriella and Georgenia. The polar lipids of strain HKI 0122^T^ consist of phosphatidylinositol and diphosphatidylglycerol together with three yet unidentified phospholipids [1].

## Genome sequencing and annotation

### Genome project history

This organism was selected for sequencing on the basis of its phylogenetic position, and is part of the Genomic Encyclopedia of Bacteria and Archaea project. The genome project is deposited in the Genomes OnLine Database [8] and the complete genome sequence in GenBank (CP001618). Sequencing, finishing and annotation were performed by the DOE Joint Genome Institute (JGI). A summary of the project information is shown in Table 2.

**Table 2:** Genome sequencing project information

MIGS ID	Property	Term
MIGS-31	Finishing quality	Finished
MIGS-28	Libraries used	Three genomic libraries: two Sanger libraries - 8 kb pMCL200 and fosmid pcc1Fos - and one 454 pyrosequence standard library
MIGS-29	Sequencing platforms	ABI3730, 454 GS FLX
MIGS-31.2	Sequencing coverage	8.56x Sanger; 10.86x pyrosequence
MIGS-30	Assemblers	Newbler version 1.1.02.15, phrap
MIGS-32	Gene calling method	Prodigal
	INSDC / Genbank ID	CP001618
	Genbank Date of Release	May 7, 2009
	GOLD ID	Gc01025
	NCBI project ID	20827
	Database: IMG-GEBA	2501416922
MIGS-13	Source material identifier	DSM 12333
	Project relevance	Tree of Life, GEBA

### Growth conditions and DNA isolation

B. cavernae HKI 0122^T^, DSM 12333, was grown in DSMZ medium 736 (Rich Medium) [13] at 28°C. DNA was isolated from 0.5-1 g of cell paste using Qiagen Genomic 500 DNA Kit (Qiagen, Hilden, Germany) with a modification of the standard protocol for cell lysis in first freezing for 20 min. (-70°C), then heating 5 min. (98°C), and cooling 15 min to 37°C; adding 1.5 ml lysozyme (standard: 0.3 ml, only), 1.0 ml achromopeptidase, 0.12 ml lysostaphine, 0.12 ml mutanolysine, 1.5 ml proteinase K (standard: 0.5 ml, only). Over night incubation at 35°C.

### Genome sequencing and assembly

The genome was sequenced using a combination of Sanger and 454 sequencing platforms. All general aspects of library construction and sequencing performed at the JGI can be found at the JGI website. 454 Pyrosequencing reads were assembled using the Newbler assembler version 1.1.02.15 (Roche). Large Newbler contigs were broken into 5,256 overlapping fragments of 1000 bp and entered into the assembly as pseudo-reads. The sequences were assigned quality scores based on Newbler consensus q-scores with modifications to account for overlap redundancy and to adjust inflated q-scores. A hybrid 454/Sanger assembly was made using the parallel phrap assembler (High Performance Software, LLC). Possible mis-assemblies were corrected with Dupfinisher or transposon bombing of bridging clones [14]. Gaps between contigs were closed by editing in Consed, custom primer walking or PCR amplification. A total of 1627 Sanger finishing reads were produced to close gaps, to resolve repetitive regions, and to raise the quality of the finished sequence. The error rate of the completed genome sequence is less than 1 in 100,000. Together all sequence types provided 19.42x coverage of the genome.

### Genome annotation

Genes were identified using Prodigal [15] as part of the Oak Ridge National Laboratory genome annotation pipeline, followed by a round of manual curation using the JGIGenePRIMP pipeline [16]. The predicted CDSs were translated and used to search the National Center for Biotechnology Information (NCBI) nonredundant database, UniProt, TIGRFam, Pfam, PRIAM, KEGG, COG, and InterPro databases. Additional gene prediction analysis and functional annotation was performed within the Integrated Microbial Genomes (IMG-ER) platform [17].

## Genome properties

**Figure 3:**
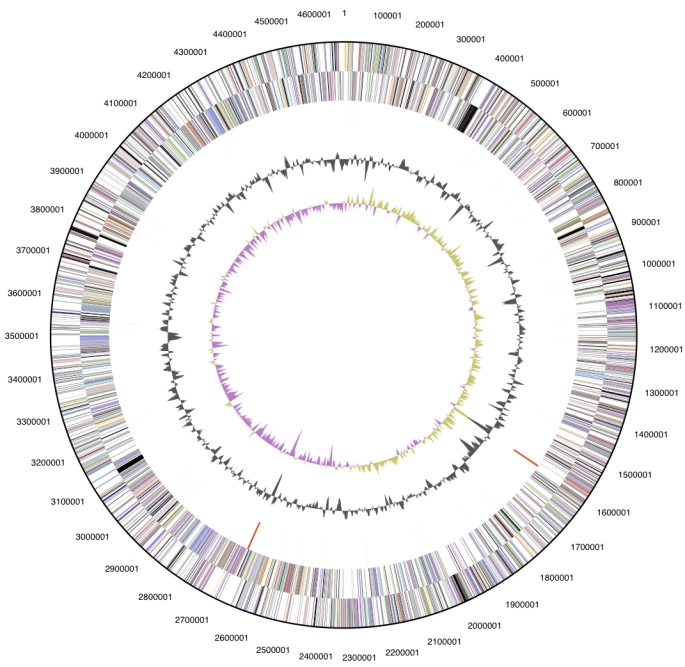
Graphical circular map of the genome. From outside to the center: Genes on forward strand (color by COG categories), Genes on reverse strand (color by COG categories), RNA genes (tRNAs green, rRNAs red, other RNAs black), GC content, GC skew

The genome is 4,669,183 bp long and comprises one main circular chromosome with a 73.1% GC content. (Table 3 and Figure 3). Of the 4278 genes predicted, 4225 were protein coding genes, and 53 RNAs. Twenty eight pseudogenes were also identified. The majority of the genes (74.3%) were assigned a putative function while the remaining ones were annotated as hypothetical proteins. The distribution of genes into COGs functional categories is presented in Table 4.

**Table 3:** Genome Statistics

Attribute	Value	% of Total
Genome size (bp)	4,669,183	100.00%
DNA Coding region (bp)	4,347,731	93.12%
DNA G+C content (bp)	3,413,947	73.12%
Number of replicons	1	
Extrachromosomal elements	0	
Total genes	4278	100.00%
RNA genes	53	1.24%
rRNA operons	2	
Protein-coding genes	4225	98.76%
Pseudo genes	28	0.65%
Genes with function prediction	3183	74.40%
Genes in paralog clusters	689	16.11%
Genes assigned to COGs	3109	72.67%
Genes assigned Pfam domains	3246	75.88%
Genes with signal peptides	1034	24.17%
Genes with transmembrane helices	1135	26.53%
CRISPR repeats	1	

**Table 4:** Number of genes associated with the 21 general COG functional categories

**Code**	**Value**	**%age**	**Description**
J	169	4	Translation, ribosomal structure and biogenesis
A	4	0.1	RNA processing and modification
K	384	9.1	Transcription
L	122	2.9	Replication, recombination and repair
B	1	0	Chromatin structure and dynamics
D	25	0.6	Cell cycle control, mitosis and meiosis
Y	0	0	Nuclear structure
V	95	2.3	Defense mechanisms
T	138	3.3	Signal transduction mechanisms
M	166	3.9	Cell wall/membrane biogenesis
N	1	0	Cell motility
W	0	0	Cytoskeleton
U	27	0.6	Extracellular structures
O	89	2.1	Intracellular trafficking and secretion
G	546	12.9	Translation, ribosomal structure and biogenesis
E	264	6.3	Carbohydrate transport and metabolism
F	92	2.2	Amino acid transport and metabolism
H	129	3.1	Nucleotide transport and metabolism
I	101	2.4	Coenzyme transport and metabolism
P	183	4.3	Lipid transport and metabolism
Q	62	1.5	Inorganic ion transport and metabolism
R	433	10.3	Secondary metabolites biosynthesis, transport and catabolism
S	249	5.9	General function prediction only
-	1116	26.4	Function unknown
